# Screening and Therapeutic Efficacy of Topical Agents for Teat Hyperkeratosis in Dairy Cows

**DOI:** 10.3390/vetsci13070608

**Published:** 2026-06-24

**Authors:** Leyao Xu, Jianfa Wang, Rui Wu

**Affiliations:** 1 College of Animal Science and Veterinary Medicine, Heilongjiang Bayi Agricultural University, Daqing 163319, China; 2 China Key Laboratory of Bovine Disease Control in Northeast China, Ministry of Agriculture and Rural Affairs, Daqing 163319, China; 3 Heilongjiang Provincial Key Laboratory of Prevention and Control of Bovine Diseases, Daqing 163319, China; 4 College of Biology and Agriculture, Jiamusi University, Jiamusi 154007, China

**Keywords:** dairy cows, teat keratinization, keratolytic agents, efficacy evaluation

## Abstract

In dairy cows, teat keratinization occurs where keratinized tissue rings form around teat tips (opening). Manifesting as skin damage in mechanically milked dairy cows, keratinization presents as dry, rough, pale, or milky-white keratinous protrusions around teat orifices and progresses via the combined effects of mechanical stress, management practices, host genetics, environmental influences, and nutritional metabolism. The teat hole is the first physiological barrier protecting the mammary glands from external pathogen invasion. Most mastitis cases are caused by pathogens invading the mammary tissues from the teat end, thereby significantly impacting mammary gland health. Thus, there are no safe, effective, economical, and standardized treatment protocols for terminal teat hyperkeratosis in dairy cows. To address this, we treated keratinization lesions using pharmacological interventions and evaluated their efficacy in 91 cows at a large commercial dairy farm in Heilongjiang Province. Urea ointment, salicylic acid ointment, and 5% azelaic acid acne cream exerted therapeutic or alleviating effects toward teat keratosis, whereas retinoic acid ointment demonstrated poor therapeutic efficacy. In a three-daily application regimen, the optimal treating dose was 0.3 g. These findings provide a scientific basis for the clinical management of hyperkeratosis in large-scale dairy farms and the development of related veterinary therapeutics.

## Simple Summary

In intensive dairy farming, terminal teat keratinization is the most common chronic lesion affecting teat tips, but it is not very noticeable in clinical settings. Currently, to ensure cleanliness, most dairy farms use teat dipping before and after milking. Teat disinfectants exert considerable antimicrobial effects, but they do not effectively target hyperkeratotic tissue on teat tips. Thus, there is a lack of safe, effective, economical, and standardized treatment protocols for terminal teat hyperkeratosis in dairy cows. In this study, we evaluated interventions targeting terminal papilla keratinization in lactating dairy cows at a large commercial dairy farm by selecting four commercial formulations (topical agents) that: (1) complied with national drug quality standards and (2) exerted unique keratolytic properties. A treatment trial was conducted on terminal papilla keratinization lesions in dairy cows to examine how well these formulations healed and improved teat health. Our aim was to identify the best treatment that improved cow health and increased milk production. In our largely descriptive study, we observed morphological alterations on cow teat tips pre- and post-administration, identified differences between groups, and determined that urea ointment was the primary treatment agent. Under a three-times-daily administration regimen, the optimal dose of urea ointment was 0.3 g.

## 1. Introduction

With the ongoing expansion in the Chinese dairy industry, the market for dairy products, a crucial dietary component, has experienced rapid growth [1]. Genetic improvements in dairy cows, along with better feeding and management approaches, have led to steady increases in milk production. The use of automated milking equipment in intensive farming systems has made farmers more productive, but concomitantly, has worsened cow welfare in terms of teat health [2]. High-vacuum milking not only causes the skin around teats to constantly rub and compress, but also disrupts normal keratin metabolism, a condition known as hyperkeratosis. When vacuum pressure is higher than 42 kPa, the teat orifice is under negative pressure. This impedes local blood circulation and damages epidermal cells [3,4,5,6,7]. A unique, coarse keratinous ring gradually develops around the orifice during extended vacuum milking; however, in extreme instances, fissures extending outwards can manifest at the orifice, resulting in characteristic “teat blooming” lesions.

Excessive teat keratinization is defined as the formation of thickened, raised keratin rings or lobes around milk duct openings. The risk to dairy cow health ranges from localized damage to systemic diseases. Teat hyperkeratosis compromises skin barrier and mammary tissue integrity; therefore, several health issues can rapidly arise when the immune defenses of the body have weakened. When excessive hyperkeratosis occurs at a teat tip, stratum corneum thickness significantly exceeds normal levels (generally 0.05–0.08 mm), resulting in reduced skin elasticity and flexibility, hardening, and increased brittleness [8]. During daily activities such as walking, lying down, or milking, this udder area is prone to cracking or damage, creating “wounds” at teat tips. These wounds lose their original protective function and become major entry routes for pathogens (e.g., *Staphylococcus aureus* and *Escherichia coli* can enter mammary tissues via the teat canals, thereby triggering mastitis [9]), and pose increasingly critical threats to mammary gland health [10,11]. Currently, intervention protocols for disinfection and sterilization at large-scale dairy farms primarily rely on pre- and post-milking teat dipping. However, these routine management practices cannot provide targeted treatments or repair localized hyperkeratotic tissue on teats.

In this study, we selected four low-cost, commercially available human topical exfoliants with proven therapeutic efficacy for cutaneous hyperkeratosis: (1) urea ointment, (2) salicylic acid ointment, (3) tretinoin cream, and (4) azelaic acid acne cream. By comparing skin conditions on the teat apex before and after treatment, we evaluated the therapeutic efficacy of these agents against teat hyperkeratosis and provided a scientific basis that may enhance teat health management and facilitate the clinical treatment of teat apex hyperkeratosis in lactating dairy cows.

## 2. Materials and Methods

### 2.1. Medicines

In accordance with the prohibited drug list for food-producing animals (Regulations on the Administration of Veterinary Topical Agents of China [12]), all test agents were screened to exclude components prohibited for use in food-producing animals [13]. We purchased 10% urea ointment from Mayinglong Pharmaceutical Group Co., Ltd. Wuhan, China (batch number: 241002); 5% salicylic acid ointment from Mayinglong Pharmaceutical Group Co., Ltd. Wuhan, China (batch number: 240102); 0.025% tretinoin (retinoic acid) cream from Hubei Kangzheng Pharmaceutical Co., Ltd. Tianmen, China (batch number: 302240402); and 5% azelaic acid acne treatment cream from Guangdong Borantang Biotechnology Co., Ltd. Zhongshan, China (batch number: 1A09A002).

### 2.2. Experimental Animals

Study cows were Holstein dairy cows with a history of continuous, stable, and high milk production, and were selected from a large-scale dairy farm in Heilongjiang Province. Dairy cows were selected based on strict inclusion and exclusion criteria and standardized feeding and management practices, and were subjected to a uniform testing protocol. In total, 91 cows were included based on their apparent health, identical feeding and management conditions, and the fact that the majority had teat end scores ≥ 3 (Skin at the teat tip was rough, with a keratinized ring 1–3 mm in diameter around the teat opening, and radial fissures extending outward from the opening, Table 1). Nearly all dairy cows received continuous treatment for teat orifice hyperkeratosis, strictly following a 20-day treatment course. Teat orifice conditions were scored and photographically documented before and after treatments.

### 2.3. Teat Scoring System

**Figure 1:**
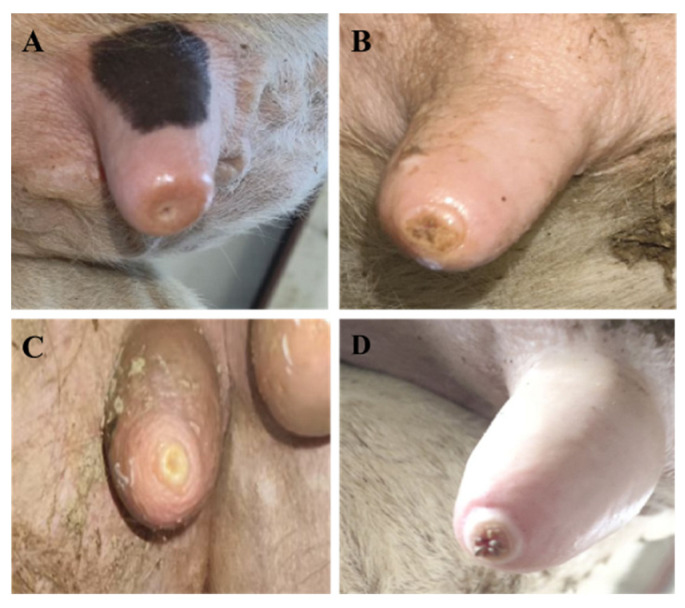
Visual references for teat-end scoring: (**A**) score = 1; (**B**) score = 2; (**C**) score = 3; (**D**) score = 4 (Table 1).

Teat scoring criteria were based on the Heilongjiang Provincial Local Standard “Technical Specifications for the Evaluation of Teat Health in Lactating Dairy Cows” [14] (See Table 1 and Figure 1). Teat orifice conditions were scored according to these standards, with real-time photographs taken (mobile phones/cameras) for documentation prior to scoring.

### 2.4. Teat Tip Scoring Method

The diagnosis and scoring of teat end hyperkeratosis primarily relied on visual and tactile evaluations by evaluators, making standardized scoring procedures essential. Standard operational protocols required assessments immediately after milking:

1. An evaluation was conducted under adequate lighting, when teats had not contracted, and keratin rings were clearly visible.
2. Palpation was applied to judge the texture of teat end keratin, which was classified as soft and elastic or hard and rough (Table 1).
3. Photographs were taken for each individual teat according to its score, rather than recording results on a per-cow basis.

### 2.5. Experimental Design

Experimental sample numbers and group allocations were determined based on scoring results. Scores were recorded for each individual teat before and after treatment, rather than documented on a per-cow basis. To investigate the therapeutic effects of experimental topical agents and evaluate their efficacy, we conducted a randomized controlled trial using urea ointment, salicylic acid ointment, retinoic acid cream, and azelaic acid acne cream. The experimental design comprised two factors: Factor A (dose) and Factor B (dosage interval). Factor A (dose) was established at four dose levels: low (0.3 g), medium (0.6 g), medium-high (0.9 g), and high (1.2 g). Factor B (dosage interval) was established at four times: every 4, 8, 12, and 24 h over a 24 h timeframe. Each formulation was tested across 16 experimental subgroups. In total, the four formulations involved 64 experimental dairy cows, enabling a comprehensive evaluation of the different dosage combinations and dosing intervals.

The lead agent, which demonstrated superior performance, was selected for further testing to determine an optimal dosage. There were treatment groups with varying topical agent dosages and a placebo control group. Twelve high-yielding dairy cows with stable long-term lactation were chosen and evenly divided into a blank control and three experimental groups. Topical cream administration was performed after each one of the three daily milking sessions. Morphological changes in teat orifices across treatment groups were observed before and after treatment, and a statistical analysis of between-group differences was conducted to identify the dosage that achieved the optimal therapeutic effect. Our results were primarily descriptive as we used subjective morphological assessments and photographic documentation.

To explore topical cream effects on physiological parameters, milk samples were collected and analyzed before and after treatment. Four separate treatment groups were established: urea ointment group, salicylic acid ointment group, retinoic acid ointment group, and azelaic acid acne cream group. In total, 12 high-yielding dairy cows, three per group, were selected during a stable, long-term lactation phase.

### 2.6. Somatic Cell Count Measurements

Before and after the trial, researchers administered the same treatment regimen to different topical agent treatment groups and analyzed somatic cell counts in milk from test cows. Before milking, the udders were wiped clean with warm, damp, disinfected towels, and the teats were disinfected again after milking. After collecting 50 mL of milk, samples were matched to cow identification numbers, and using a somatic cell counter, milk parameters were measured and recorded.

### 2.7. Blindly Evaluating Teat Characteristics

Teat photographs from experimental animals were organized to cover three key time points across the trial: before treatment, Day 10, and Day 20. Images were anonymized by standardized cropping and formatting, background information removal, and assigning anonymous filenames.

Two independent evaluators were recruited, both professionally qualified in teat hyperkeratosis scoring, and importantly, were free from subjective bias. In strict accordance with the guidelines [14], scoring thresholds were unified, and consistent criteria were established for grading keratin ring thickness, surface roughness, and cracking severity at all score levels. In total, 100 teat photos were randomly selected for preliminary scoring. Discrepant scores were jointly discussed to calibrate rating criteria. Formal scoring commenced only after the inter-rater reliability score reached a Kappa coefficient of ≥0.75, indicating substantial agreement between evaluators.

### 2.8. Statistical Analysis

Experimental data were sorted and summarized using WPS Office 12.1.0 software. Data were analyzed by one-way ANOVA and paired *t*-tests, and graphs were assembled in GraphPad Prism 9. Results were expressed as the mean ± standard deviation (mean ± SD). In figures and tables, “ns” indicated a non-significant difference (*p* > 0.05) and * indicated a significant difference (*p* < 0.05).

## 3. Results

### 3.1. Morphological Changes in the Blank Control Group

**Figure 2:**
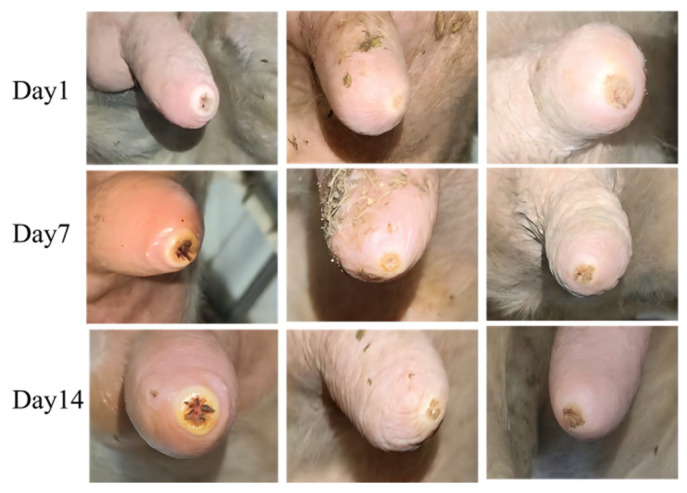
Morphological changes at teat tips in the blank control group.

To eliminate the impact of non-drug factors such as milking management and environmental changes on the experimental results. To determine the clinical value of topical creams, we established an untreated control group (three dairy cows with severely prominent teat hyperkeratosis). After a 2-week observation period, teats exhibited rough skin at the tip, and radial cracks radiating outward from the teat orifice, and the keratin ring on the surface had become increasingly prominent, while neither keratin protrusions nor cracks had shown any relief/improvement (Figure 2). Thus, keratinization issues did not spontaneously resolve over time.

### 3.2. Score Changes Across Treatment Groups

The urea group exhibited the best therapeutic effects. All urea dosages reduced teat end scores by 2 points. Salicylic acid ranked second: the low-dose group (0.3 g) achieved a 1-point reduction; the medium-dose group (0.6 g) generated reductions of 2 points and 1 point; and the medium-high dose group (0.9 g) decreased scores by 1 point under both 4 h and 8 h medication intervals. For the tretinoin group, low (0.3 g), medium (0.6 g), and medium-high (0.9 g) doses only reduced scores by 1 point. In the azelaic acid group, the low-dose treatment (0.3 g) with 4 h and 8 h dosing intervals lowered scores by 2 points; medium (0.6 g) and medium-high (0.9 g) doses reduced scores by 1 point, while the high dose group (1.2 g) achieved a 2-point reduction. Score changes across treatment groups are shown (Table 2, Table 3, Table 4 and Table 5).

### 3.3. Morphological Changes in the Urea Treatment Group

**Figure 3:**
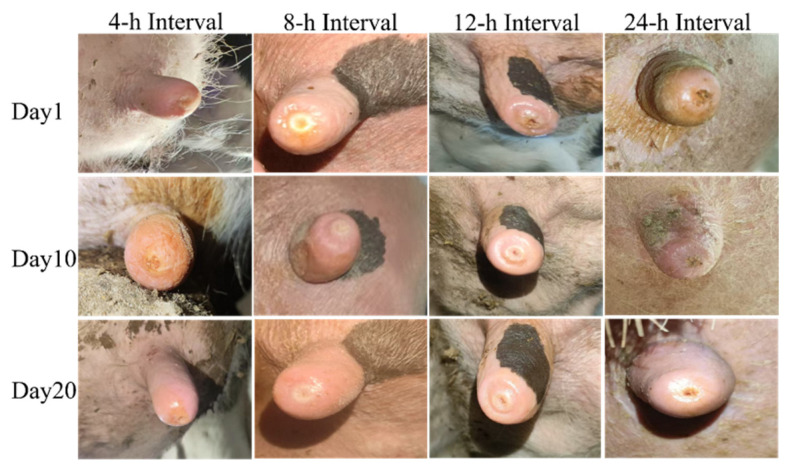
Morphological changes in teat apex orifices on days 1, 10, and 20 following low-dose urea administration.

The urea treatment group achieved excellent therapeutic effects at low doses (Figure 3). When a low-dose urea treatment was administered at 4 h intervals from day 20, teats exhibited a rounded, plump appearance with a smooth surface, indicating good therapeutic effects at low urea doses.

### 3.4. Morphological Changes in the Salicylic Acid Treatment Group

**Figure 4:**
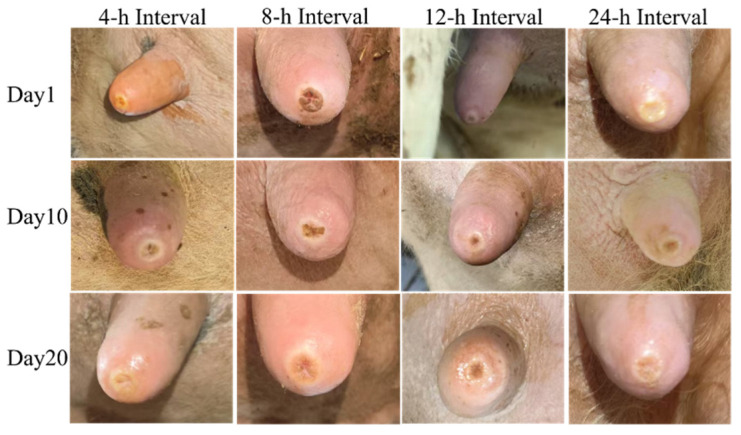
Morphological changes in teat apex orifices on days 1, 10, and 20 following medium-dose salicylic acid administration.

Morphological changes in teat orifices at medium salicylic dose levels on days 1, 10, and 20 are shown (Figure 4). By day 20 of continuous treatment, teats receiving medium doses at 4 h and 8 h intervals showed significant morphological recovery at teat ends, whereas for teats receiving 12 h and 24 h applications, teat scores only recovered to 2 points. Thus, salicylic acid at medium doses and above, when applied at 4 h and 8 h intervals, achieved good therapeutic effects.

### 3.5. Morphological Changes in the Retinoic Acid Treatment Group

**Figure 5:**
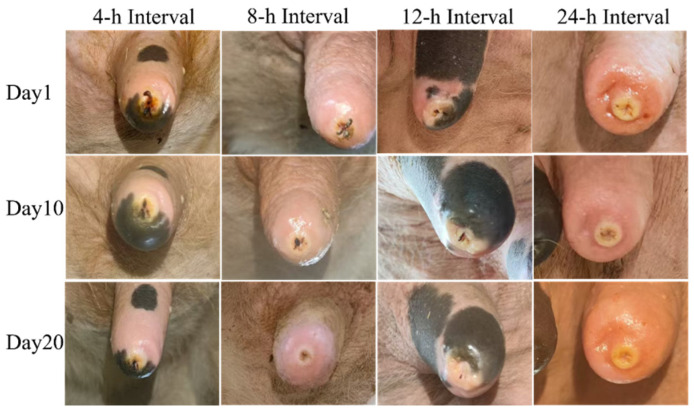
Morphological changes in dairy cow teat apex orifices on days 1, 10, and 20 following medium-high tretinoin doses.

Across the four medium-high dose tretinoin groups with 4 h, 8 h, 12 h, and 24 h application intervals, no cow teat showed a smooth skin surface with a flat, small circular orifice at the teat apex. By day 20 of treatment, the medium-high dose groups with 4 h and 8 h application intervals had only improved teat scores from 4 to 3 (Figure 5). Among all topical agents, the tretinoin treatment group exhibited the poorest therapeutic efficacy over the 20-day continuous administration period.

### 3.6. Morphological Changes in the Azelaic Acid Treatment Group

**Figure 6:**
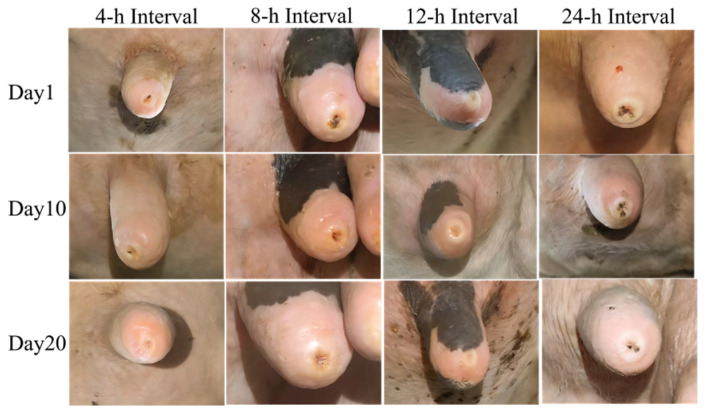
Morphological changes in teat apex orifices on days 1, 10, and 20 following medium azelaic acid doses.

The azelaic acid treatment group did not demonstrate any significant therapeutic effects. At moderate doses and 4 h and 8 h dosing intervals, azelaic acid produced relatively pronounced changes in keratinized tissue at teat tips, with initial teat tip scores reducing from 4 to 2/3 (Figure 6).

Due to healing processes in milk duct openings, keratinized tissue accumulates at teat tips. Keratin layer treatment and resolution using keratolytic agents is a relatively lengthy process, characterized by “keratin dissolution, keratin breakdown, keratin proliferation, keratin drying, and keratin shedding” stages.

### 3.7. Teat Score Changes in Determining Optimal Urea Doses

For both dosage and application intervals, all four topical formulations were more or less effective in treating/alleviating teat keratosis symptoms. Salicylic acid was the second most effective treatment, followed by urea ointment. Azelaic acid acne ointment showed moderate efficacy in treating symptoms, while retinoic acid ointment was the least effective.

**Figure 7:**
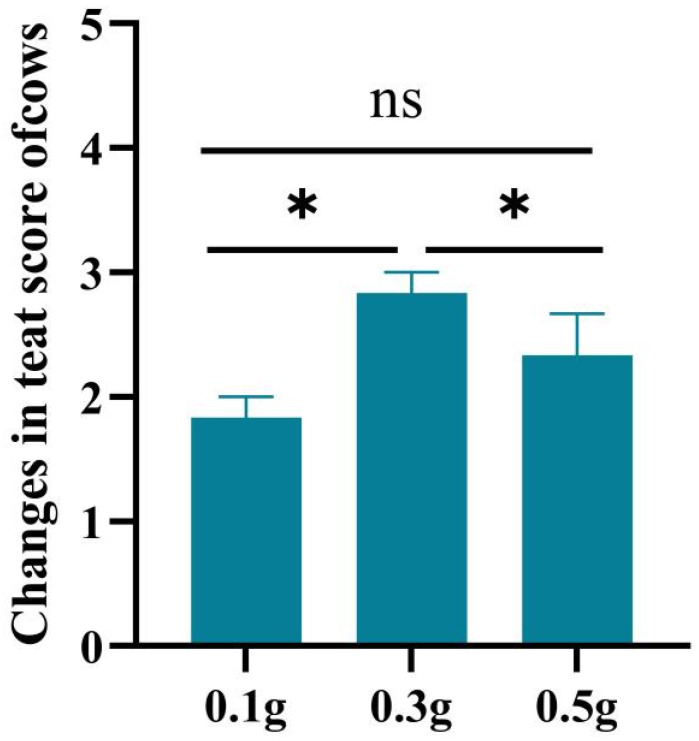
Teat score changes during optimal urea dosage screening. Significant teat score differences were recorded between the 0.3 g group and the 0.1 g and 0.5 g groups; however, no significant difference was recorded between the 0.1 g and 0.5 g groups. The chart shows standard deviation values for *n* = 3 cows. One-way ANOVA and paired *t*-tests were used (* *p* < 0.05). ns = not significant.

The optimal dosage range for urea ointment was established between low-dose (0.3 g) and medium-dose groups (0.6 g), with both demonstrating relatively good efficacy. To facilitate three daily milking sessions, the medication was administered thrice daily, post-milking. By observing morphological changes in keratinized tissue at teat tips before and after treatment, and conducting statistical analyses, the optimal therapeutic urea dose, over a three-times-daily administration regimen, was 0.3 g (Figure 7).

### 3.8. Topical Agent Effects on Somatic Cell Counts

**Figure 8:**
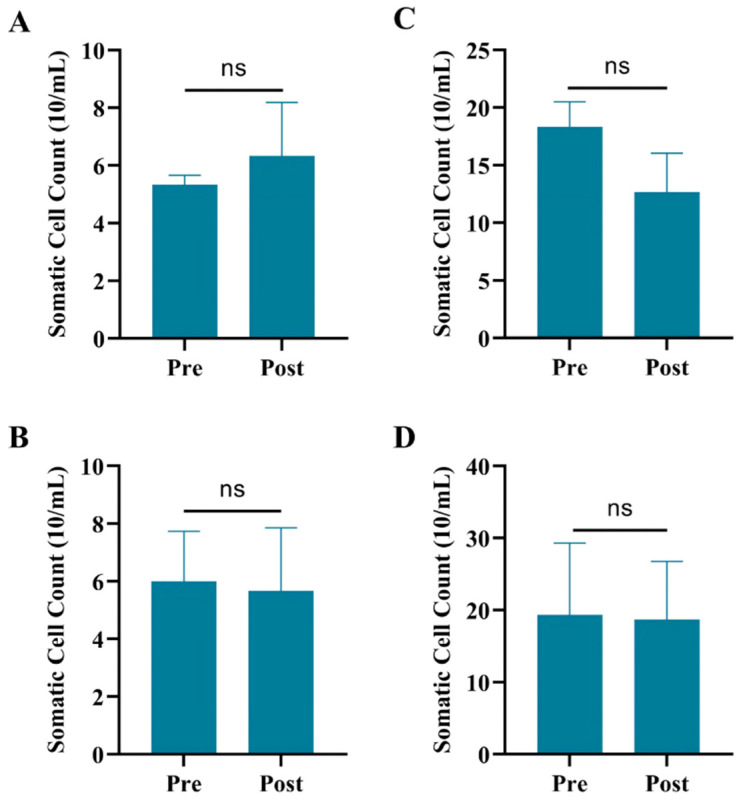
Graphs show animal groupings prior to (pre) and subsequent to (post) topical agent administration (horizontal axis), while the vertical axis denotes somatic cell counts. Milk samples were obtained from the same cows in groups prior to and following topical agent administration, with somatic cell counts undergoing statistical analysis. (**A**) Urea treatment cohort, (**B**) salicylic acid treatment cohort, (**C**) azelaic acid treatment cohort, (**D**), and retinoic acid treatment cohort. Standard deviation values are shown for *n* = 3 cows. One-way ANOVA and paired *t*-tests were used (ns = non-significant, *p* > 0.05).

Variations in somatic cell counts can reflect cow health. In our analyses, somatic cell counts across groups were consistent (Figure 8); for all topical agents, no effects on somatic cell counts were recorded, suggesting that treatments did not influence the health status of study cows.

## 4. Discussion

Currently, domestic dairy farms primarily rely on iodine- and chlorine-based teat dips for teat care before and after milking. Although these formulations provide broad-spectrum disinfection, they do not provide targeted treatment or repair for existing hyperkeratotic tissue. Currently, there is a lack of safe, effective, economical, and standardized treatment protocols for terminal teat hyperkeratosis in dairy cows. With ongoing structural adjustment and optimization in the Chinese dairy cattle industry, and continuous improvements in intensive, barn-fed dairy farming, the gradual replacement of traditional manual milking by mechanical milking has become inevitable [15]. However, as a highly prevalent clinical condition in intensive dairy farming, excessive teat keratinization reduces milking efficiency and increases teat orifice injury risks during milking, and it also compromises teat skin barrier function. Additionally, cold winter weather maintains teat tip shape in a constant state of flux [16,17]. Teat end conditions in a herd can change considerably in just a few days, particularly when the weather is unfavorable or the climate changes quickly. Studies have reported that teat end scores are linked to a higher risk of mastitis infection, and that keratinization levels at teat ends are strongly linked to mastitis incidences [18].

Following the topical application of four test agents (urea ointment, salicylic acid ointment, tretinoin cream, and azelaic acid acne cream) to keratinized teat tissues, therapeutic effects became progressively apparent. A 20-day follow-up study of a subset of animals in the trial protocol after discontinuation of the drug showed that all nipple tip scores had increased (i.e., the severity of the lesions had worsened). In other words, over the trial duration, symptoms improved, scores decreased during active treatment, but after treatment cessation, the teat apex returned to a rough state with prominent keratin rings. Thus, preventing and treating teat keratinization is a complex and long-term management task.

Previous studies have indicated that the urea in urea ointment effectively hydrates and exfoliates dead skin cells. It prevents the stratum corneum from obstructing follicular orifices [19], thereby improving keratotic lesions. Salicylic acid ointment contains salicylic acid, which degrades keratin, facilitates dead skin desquamation from the stratum corneum, eliminates excessive stratum corneum thickness, and accelerates epidermal metabolism [20]. Azelaic acid acne cream is a topical antibacterial agent that exerts anti-keratinizing effects on hair follicle epithelium and inhibits protein synthesis; however, in our high-dose azelaic acid treatment group, keratinized tissue at papillary pores fluctuated between destruction and hyperplasia, preventing optimal therapeutic outcomes [21]. In contrast, the 0.025% retinoic acid cream used here is a low-concentration formulation for facial acne in humans. At this concentration, transdermal absorption is insufficient because the keratolytic effects of retinoids depend on regulated epidermal cell proliferation and differentiation; thus, the 20-day treatment period was possibly insufficient to observe optimal efficacy, and possibly failed to reach an effective concentration for regulating keratinocyte differentiation. However, the treatment duration was also insufficient [22], because the keratolytic effects of retinoids depend on the regulation of epidermal cell proliferation and differentiation, so the 20-day treatment period in this study may not have been sufficient to observe their optimal efficacy.

From a clinical mechanism perspective, high-quality, topical teat preparations should exert multiple integrated effects, including moisturizing and softening keratin, anti-inflammatory soothing, and promoting epithelial repair and bacteriostasis for wound protection. They can abnormally soften hyperplastic keratin and facilitate the orderly shedding of aged cuticles, thereby preventing teat deformation and poor orifice closure caused by accumulated and hardened keratin. Such preparations repair damaged cutaneous epithelial tissues and reconstruct the physical skin barrier of the teat, enabling resistance to persistent irritation from environmental microflora, mechanical friction, and negative milking pressure, thereby fundamentally reducing teat injury and mammary gland infections. When compared with conventional, simplistic management approaches, such as single petrolatum moisturization or routine disinfectant applications, the specialized topical agents used in this study showed better targeting performances, superior repair efficiency, and lower irritation effects. They were better adapted to the delicate physiological characteristics of bovine teats that ensure long-term mechanical stress, and therefore demonstrated considerable advantages in terms of therapeutic applications.

Teat hyperkeratosis generates significant economic losses for dairy farmers. The condition increases treatment costs and also leads to a series of negative effects, including reduced milk production, decreased high-quality milk supply, and diminished farming profitability. In our systematic topical agent screen and scientific evaluation, we determined the efficacy of different medications toward teat hyperkeratosis. This work could allow animal health product manufacturers to develop suitable, specialized medicated bath solutions for teat hyperkeratosis, thereby helping farmers to effectively prevent mastitis, improve milk yields and milk quality, and increase farming profits [23].

## 5. Conclusions

We showed that urea ointment, salicylic acid ointment, and azelaic acid acne cream provided therapeutic or alleviating effects toward teat hyperkeratosis in dairy cows, whereas tretinoin ointment demonstrated limited therapeutic efficacy. The ideal dosage for treating the condition was 0.3 g, with three applications/day. Our study provides a scientific basis for the clinical management and the advancement of veterinary therapeutics in treating terminal teat hyperkeratosis in dairy cows.

## Figures and Tables

**Table 1:** Teat end scoring criteria.

Score	Teat End Callus Ring Thickness
1	The skin at the tip of the teat is smooth and has a small, flat opening.
2	The tip of the teat has a smooth keratinous ring approximately 1 mm thick.
3	The skin at the tip of the teat is rough, with a keratinized ring 1–3 mm in diameter around the teat opening, and radial fissures extending outward from the opening.
4	The teat orifice has a very rough, keratinized ring measuring more than 3 mm in diameter, and there are noticeable fissures extending outward from the teat orifice.

**Table 2:** Teat tip scores for the urea group: before medication, day 10, and day 20.

Dosage Regimens	Dosing Interval	Before Treatment	Day 10 of Treatment	Day 20 of Treatment
Low-dose group (0.3 g)	4 h	3	2	1
	8 h	3	2	1
	12 h	3	2	1
	24 h	3	2	1
Medium-dose group (0.6 g)	4 h	4	3	1
	8 h	4	4	2
	12 h	3	2	1
	24 h	4	4	2
Medium-to-high dose group (0.9 g)	4 h	3	2	1
	8 h	3	3	1
	12 h	4	3.5	2
	24 h	4	3	2
High-dose group (1.2 g)	4 h	3	1	1
	8 h	3	2	2
	12 h	4	2	2
	24 h	4	3	2

**Table 3:** Teat tip scores for the salicylic acid group: before medication, day 10, and day 20.

Dosage Regimens	Dosing Interval	Before Treatment	Day 10 of Treatment	Day 20 of Treatment
Low-dose group (0.3 g)	4 h	3	2	2
	8 h	4	4	3
	12 h	3	2	2
	24 h	4	4	3
Medium-dose group (0.6 g)	4 h	3	2	1
	8 h	4	4	2
	12 h	4	2	2
	24 h	3	3	2
Medium-to-high dose group (0.9 g)	4 h	3	3	2
	8 h	3	2	2
	12 h	3	3	2
	24 h	3	4	3
High-dose group (1.2 g)	4 h	4	3	1
	8 h	3	2	1
	12 h	4	3	2
	24 h	4	4	3

**Table 4:** Teat tip scores for the retinoic acid group: before medication, day 10, and day 20.

Dosage Regimens	Dosing Interval	Before Treatment	Day 10 of Treatment	Day 20 of Treatment
Low-dose group (0.3 g)	4 h	3	2	2
	8 h	3	3	2
	12 h	4	4	3
	24 h	4	3	3
Medium-dose group (0.6 g)	4 h	4	3	3
	8 h	4	3	3
	12 h	4	4	3
	24 h	4	4	3
Medium-to-high dose group (0.9 g)	4 h	4	4	3
	8 h	4	4	3
	12 h	4	3	3
	24 h	3	3	3
High-dose group (1.2 g)	4 h	3	3	2
	8 h	3	3	2
	12 h	3	3	2
	24 h	4	3	2

**Table 5:** Teat tip scores for the azelaic acid group: before medication, day 10, and day 20.

Dosage Regimens	Dosing Interval	Before Treatment	Day 10 of Treatment	Day 20 of Treatment
Low-dose group (0.3 g)	4 h	4	3	2
	8 h	4	3	2
	12 h	4	4	3
	24 h	4	4	3
Medium-dose group (0.6 g)	4 h	3	3	2
	8 h	4	3	3
	12 h	3	3	2
	24 h	4	4	3
Medium-to-high dose group (0.9 g)	4 h	3	3	2
	8 h	4	4	3
	12 h	3	3	2
	24 h	4	3	3
High-dose group (1.2 g)	4 h	4	4	2
	8 h	4	3	2
	12 h	4	4	2
	24 h	4	4	3

## Data Availability

The original contributions presented in this study are included in the article. Further inquiries can be directed to the corresponding authors.
